# Enhanced electrochemical performance of the MoS_2_/Bi_2_S_3_ nanocomposite-based electrode material prepared by a hydrothermal method for supercapacitor applications

**DOI:** 10.1039/d3ra03892k

**Published:** 2023-08-14

**Authors:** Kamal Batcha Mohamed Ismail, Manoharan Arun Kumar, Ramasamy Jayavel, Mukannan Arivanandhan, Mohamed Abubakkar Mohamed Ismail

**Affiliations:** a Department of Electrical, Electronics & Communication Engineering, School of Technology, Gandhi Institute of Technology and Management (GITAM) Bengaluru-561 203 India manokavi2011@gmail.com mohamedismailmk@gmail.com +91-7708587758; b Department of Electronics & Communication Engineering, Agni College of Technology Chennai-600 130 Tamil Nadu India; c Centre for Nanoscience and Technology, Anna University Chennai-600 025 Tamil Nadu India

## Abstract

Supercapacitors are widely used energy storage systems in the modern world due to their excellent electrochemical performance, fast charging capability, easy handling, and high power density. In the present work, pure MoS_2_ and MoS_2_/Bi_2_S_3_ nanocomposites with different compositions of bismuth were synthesized by the hydrothermal method. The structural properties of the electrode materials were studied using the XRD technique, which confirmed the formation of MoS_2_ and the secondary phase of Bi_2_S_3_ while increasing Bi substitution. The morphological studies of the synthesized electrode materials were performed using SEM, TEM, and HRTEM techniques, which indicated the 3D layered hierarchical structure of MoS_2_ nanospheres and the nanosheet-like structure of Bi_2_S_3_. The electrochemical properties of pristine MoS_2_ and MoS_2_/Bi_2_S_3_ nanocomposites were analysed by CV, CP, and EIS techniques using a 2 M KOH electrolyte in a three-electrode system. The CV curves show evidence of significant improvement in the electrochemical performance of MoS_2_/Bi_2_S_3_ composites compared to that of pure MoS_2_. The calculated specific capacitances of MoS_2_/Bi_2_S_3_ nanocomposites were relatively higher than those of pristine MoS_2._ The 20 mol% Bi added sample showed a maximum specific capacitance of 371 F g^−1^, compared to pristine MoS_2_ and other samples at a current density of 1 A g^−1^. The kinetics of the electrochemical process was studied. The Nyquist plots indicated that the Bi-added nanocomposites had lower *R*_esr_ and *R*_CT_ values, which resulted in high electrochemical performance. The experimental results revealed that Bi-substitution can further enhance the electrochemical energy storage performance of MoS_2_ for supercapacitor applications.

## Abstract 2

Supercapacitors are widely used energy storage systems in the modern world due to their excellent electrochemical performance, fast charging capability, easy handling, and high power density.

## Graphical abstract

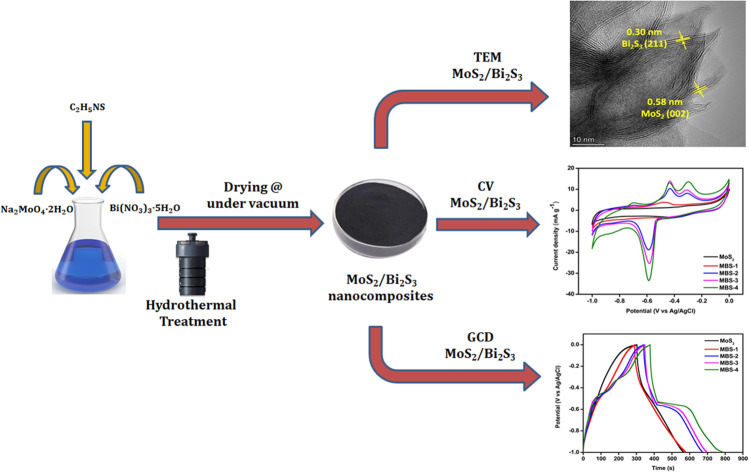

## 1. Introduction

Recent studies in nanotechnology have paved the way for designing and preparing various nanostructured materials. These materials have the potential to revolutionize numerous industries by offering improved performance, new functionality, and unprecedented control over material properties. Nanostructured materials will dominate future energy storage and conversion technology, particularly supercapacitors, Li-ion batteries, thermoelectrics, and photovoltaic applications.^1–4^ The supercapacitor is remarkably used as energy storage devices because of their capability to achieve high energy density than typical capacitors and high power density than Li-ion batteries. Typically, supercapacitors bridge the gap between conventional capacitors and batteries, which offers high capacitance, fast charging, slow discharging, more extended life cycles, high stability, superior performance at low temperatures, less weight, and cost-effectiveness. The storage capacity of supercapacitors is primarily determined by the surface area and pore structure of electrodes, which affects the available electrode–electrolyte interface for charge storage. Supercapacitors excel in applications requiring rapid energy transfer and high power output. The high self-discharge rate and lower energy density compared to batteries are the challenges in supercapacitors, which restrict their lower voltage applications.^5,6^

Supercapacitors store energy through two behaviours, such as electric double-layer capacitance (EDLC) and pseudo capacitance. In EDLC supercapacitors, the electrode material stores the charges through electrostatic adsorption and desorption of electrolyte ions. At the surface of electrodes, there is no electrical charge transfer between electrodes and electrolytes.^7,8^ EDLC supercapacitors have high power density and good cyclic stability. Low-dimensional carbon-based materials such as graphene, carbon nanotubes, graphene oxides, and reduced graphene oxides have good EDLC behaviour.^9–12^ Pseudocapacitance is an electrochemical phenomenon where the existence of fast sequences of adsorption and intercalation charge transfers with reversible structural modifications resulted in high charge storage capacity. The fast electron charge transfer takes place due to a reversible oxidation and reduction reaction on the surface of the electrode and electrolyte interface, which produces a faradaic capacitive type current. Conducting polymers, metal oxides, and metal sulphides have delivered a profound pseudocapacitive behaviour.^13^ The specific capacitance of these electrochemical capacitors is 10–100 times greater than carbon-based electrode materials for supercapacitor applications.^14^

Various kinds of nanostructured electrode materials are employed in supercapacitor applications, wherein, two-dimensional layered materials have a large surface area, which allows maximum active sites on the surface, more flexibility, and high chemical and thermal stability. Hence, 2D materials are highly suitable as electrodes for electrochemical capacitors. Transition metal dichalcogenides (TMDs) have good catalytic and electrochemical performance because of their high conductivity among various 2D materials beyond graphene.^15,16^ The general formulas for TMDs are AB_2_, where the term A refers to transition metals, and the term B refers to chalcogen elements such as sulphur, selenium, and tellurium. In the crystal structure of TMDs, the layers of transition metal atoms were sandwiched between the layers of chalcogen atoms.^17^ The majority of TMDs have the capacity to exist in more than one crystal structure, notably the semiconducting (2H) and semimetal (1T) phases, and have physical and chemical properties that vary with thickness. Each layer in the TMD structures is weakly bonded to its neighbours by van der Waals forces, making physical or chemical isolation simple.^18^ MoS_2_ belongs to the family of TMDs, which have a large surface area, rich redox peaks, good intrinsic properties, and high theoretical capacitance. These properties of MoS_2_ make it suitable for energy storage applications such as supercapacitors.^19–21^ The monolayer MoS_2_ has a direct band gap of 1.8 eV, and the bulk MoS_2_ has an indirect band gap is about 1.2 eV. The layered structure of molybdenum disulfide is similar to graphene, which supports the fast moving of electrons in the redox reactions.^22–25^

Bismuth materials are used in numerous applications such as gas sensors, catalysts, and energy storage systems because of their low cost, nontoxic properties, high conductivity, and dielectric behaviour. Bismuth-based nanocomposite materials having relatively good redox reactions in the negative potential window can be used as negative electrodes in energy storage systems.^26,27^ Bismuth compounds such as bismuth oxides and bismuth sulfides are n-type semiconductors having high refractive index, narrow energy band gap, and good electrochemical activities. The surface morphology, crystalline structure, size, shape, and dimensions of bismuth chalcogenides have a significant impact on their electrochemical properties.^28^ Due to the substantial intercalation space in their crystalline structure, bismuth chalcogenides are used in electrochemical energy storage devices; their lattice interlayer provides routes for the transit and employment of guest ions.^29^ The inclusion of other molecules into MoS_2_ nanosheets can increase the layer distance between each stacked layer, which impacts the electrochemical behaviour. The metal oxide and sulfide-based MoS_2_ hybrid electrodes have enhanced electrochemical performance because of their high pseudocapacitance, which can store charge at a higher rate than a conventional electric double-layer capacitor.^30,31^

A viable option is to combine a nanomaterial with metal sulfides in order to address the conductivity limitations of individual metal sulfides. This integration offers flexible platforms for effective charge transfer. MoS_2_, Bi_2_S_3_, CuS, NiS, NiS_2_, Ni_3_S_2_, CoS, CoS_2_, CdS, SnS, and SnS_2_ are all promising choices for supercapacitor electrodes due to their impressive electrochemical performances.^32–36^ S. Jia *et al.* developed a composite material NiCo-DHS, which exhibited a high specific capacity of 973.6C g^−1^ and remains durable over 8000 cycles, with only a small capacity loss of 7.4%. It achieved an energy density of 65.91 W h kg^−1^ and a power density of 0.89 kW kg^−1^, demonstrating its considerable potential.^37^ A nonaqueous sodium-ion hybrid energy storage device has been developed, which utilizes VS_2_ nanosheets grown on electrochemically exfoliated graphene as the negative electrode and activated carbon as the positive electrode. These hybrid devices exhibited a high areal capacitance of 110.7 mF cm^−2^ over a wide potential range of 0.01–3.5 V. Additionally, they demonstrated an impressive areal energy density of 188.3 μW h cm^−2^ and excellent cycling stability, maintaining their performance for up to 5000 cycles without noticeable decay.^38^ W. Zhang *et al.* developed a Ti_3_C_2_Tx/Bi_2_S_3_@N-C electrode that showed a specific capacitance of 653 F g^−1^, which is significantly higher than other binary composites or similar MXene-based materials. The Zinc-ion hybrid capacitor consists of Ti_3_C_2_Tx/Bi_2_S_3_@N-C and Zn foil as electrodes, and it exhibits a high specific capacitance of 150.33 F g^−1^, along with a maximum energy density of 46.98 W h kg^−1^ at a power density of 750 W kg^−1^.^39^ Utilizing metal sulfides in supercapacitors offers numerous benefits, including high energy density, improved stability, enhanced conductivity, a wide range of materials, environmental advantages, compatibility with flexible devices, and potential for hybrid systems. This makes them an attractive choice for advancing the field of supercapacitor technology. However, only a few studies have been published so far, on the effects of Bi-substitution in MoS_2_ for energy storage applications. Therefore, the main objective of the present work is to study the effects of Bi-substitution in MoS_2_/Bi_2_S_3_ nanocomposite materials on their structural, morphological, and electrochemical performance.

## 2. Materials and methods

### 2.1. Materials

Sodium molybdate dehydrate (Sigma-Aldrich laboratory with 99.5% purity), thioacetamide (Alfa Aesar laboratory with 98% purity), and bismuth nitrate (Alfa Aesar laboratory with 98% purity) were used as precursors. All chemicals and reagents were purchased as analytical grade and used without any further purification.

### 2.2. Hydrothermal synthesis of pure MoS_2_

The hydrothermal method was used to synthesize pure MoS_2_ nanoparticles. First, 0.05 M of sodium molybdate dihydrate (Na_2_MoO_4_·2H_2_O) and 0.1 M of thioacetamide (C_2_H_5_NS) were taken in the molar ratio of 1 : 2. The precursors were dissolved in 140 ml DI water and stirred for 30 minutes. Then, the solution was transferred into a 200 ml Teflon-lined stainless steel autoclave. After that, the autoclave was kept in the box furnace at 200 °C for 48 hours. The black precipitate was collected and washed several times using ethanol and deionized water. Finally, the sample was dried in a vacuum oven at 60 °C for 6 hours.

### 2.3. Hydrothermal synthesis of MoS_2_/Bi_2_S_3_ nanocomposites

**Fig. 1:**
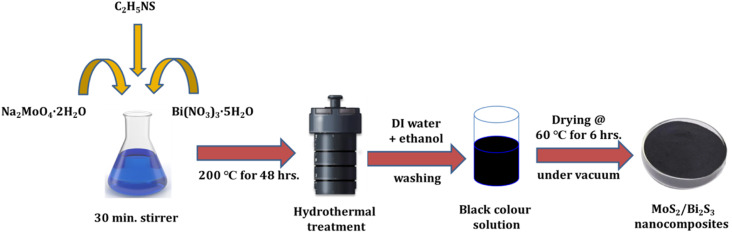
Schematic representation of the hydrothermal synthesis of MoS_2_/Bi_2_S_3_ nanocomposites.

MoS_2_/Bi_2_S_3_ nanocomposites were synthesized using a hydrothermal method by adding Bi with different mol%. To synthesize 5 mol% Bi-added composite, 0.0475 M of sodium molybdate dihydrate, 0.1 M of thioacetamide, and 0.0025 M of bismuth nitrate were dissolved in 140 ml of DI water and further stirred for 30 minutes. The resultant solution was transferred to a Teflon-lined stainless steel autoclave and kept in a box furnace at 200 °C for 48 h. Finally, the black color precipitate was collected and washed out several times using ethanol and deionized water and then dried in a vacuum oven at 60 °C for 6 h. A similar procedure was followed to synthesize composites with different Bi concentrations of 5, 10, 15, and 20 mol% and the samples were named as MBS-1, MBS-2, MBS-3, and MBS-4, respectively. A schematic representation of the hydrothermal synthesis of MoS_2_/Bi_2_S_3_ nanocomposites is shown in Fig. 1.

### 2.4. Characterization techniques

The morphology of pristine MoS_2_ and MoS_2_/Bi_2_S_3_ nanocomposites was analyzed using the TESCAN VEGA 3 Scanning electron microscope (SEM) and FEI TECHNAI G2 20 TWIN high-resolution transmission electron microscope (HRTEM). The structural properties of the pristine MoS_2_ and MoS_2_/Bi_2_S_3_ nanocomposites were analyzed by XRD using a REGAKU SMARTLAB diffractometer. The XPS analysis of pristine MoS_2_ and MoS_2_/Bi_2_S_3_ nanocomposites was performed on a PHI Versaprobe III instrument. The electrochemical properties of pristine MoS_2_ and MoS_2_/Bi_2_S_3_ nanocomposites were studied by CP, CV, and EIS analysis using CH Instruments, USA (CHI6008E).

### 2.5. Electrochemical studies

The active electrode material, Super P® carbon black and polyvinylidene fluoride (PVDF) were taken in the weight ratio of 80 : 10 : 10 respectively, and well mixed using a mortar and pestle. The slurry was prepared from the above mixture with *N*-methylpyrrolidone (NMP) as a solvent. Super P® carbon black was used as a conductive additive and PVDF was used as a binder. The prepared slurry was coated on Ni foam over the area of 1 cm^2^, which served as the working electrode in a three-cell electrode setup. The total weight of the slurry coated on one working electrode was 4 mg, and the areal mass loading of the active electrode material was 3 mg. A platinum wire and Ag/AgCl served as counter and reference electrodes, respectively. 2 M KOH was used as the electrolyte in the electrochemical studies of pristine MoS_2_ and MoS_2_/Bi_2_S_3_ nanocomposites. The electrochemical properties were analyzed using CH Instruments at room temperature. The cyclic voltammetry (CV), chronopotentiometry (CP), and electrochemical impedance spectroscopy (EIS) tests were carried out using a three-cell electrode configuration.

## 3. Results and discussion

### 3.1. Structural and morphological analysis

**Fig. 2:**
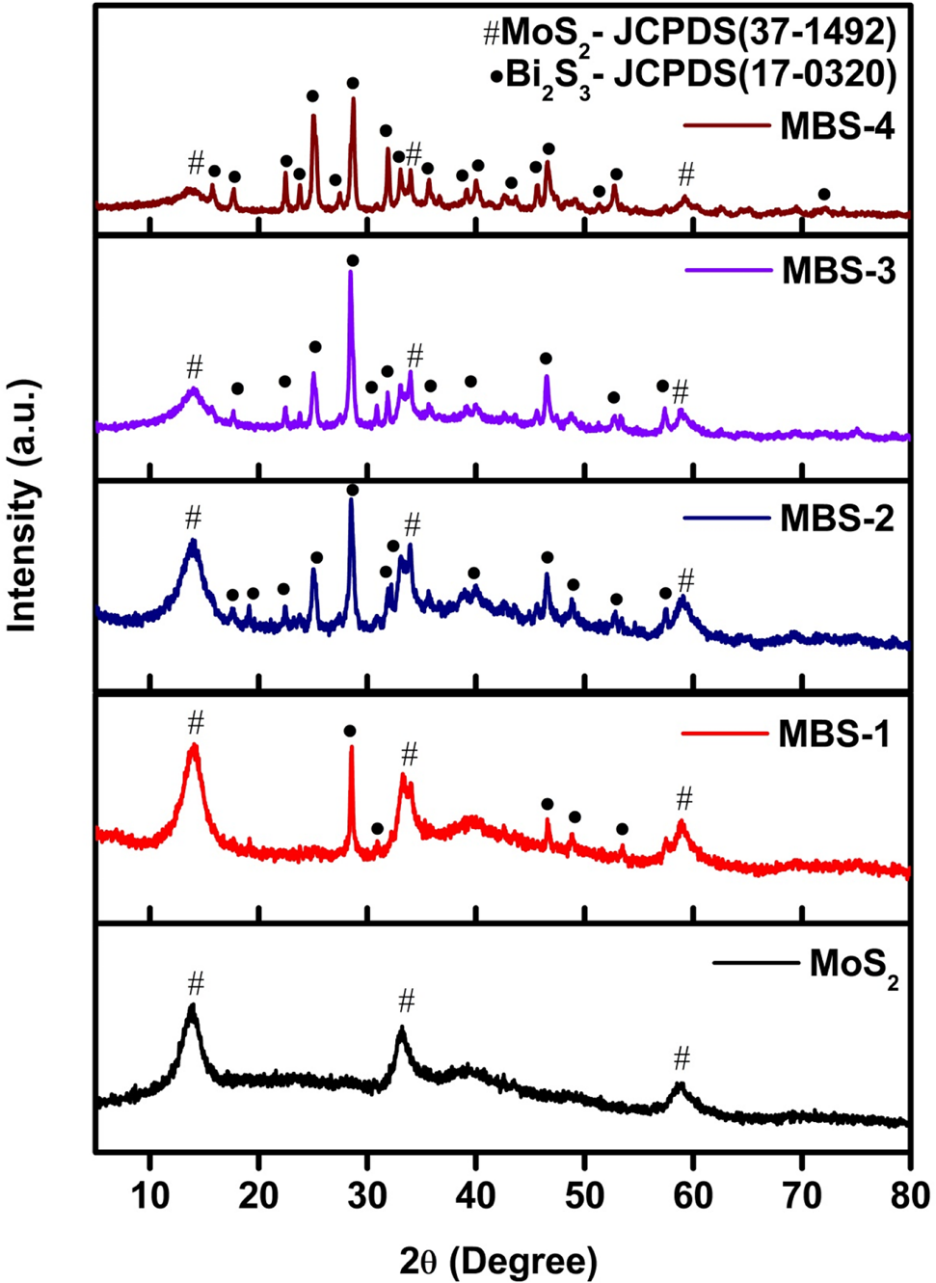
XRD patterns of the pristine MoS_2_, MBS-1, MBS-2, MBS-3, and MBS-4 samples.

XRD patterns of pristine MoS_2_ and MoS_2_/Bi_2_S_3_ nanocomposites recorded in the 2*θ* range from 5–80° and shown in Fig. 2. The diffracted peaks of pristine MoS_2_ indexed to the planes of (002), (100), and (106) well matched with the standard JCPDS data (card no. 37-1492).^40^

The secondary phase of Bi_2_S_3_ was formed by increasing the Bi concentration in MoS_2_/Bi_2_S_3_ nanocomposites. The diffraction peak intensities of Bi_2_S_3_ were indexed to the planes of (200), (120), (220), (101), (310), (211), (221), (240), (430), (440), (501), (312), (360), and (811) were in good agreement with those from the standard JCPDS data (card no. 17-0320). The mixed phases of MoS_2_ and Bi_2_S_3_ were clearly observed in the XRD patterns of MoS_2_/Bi_2_S_3_ nanocomposites and no other diffraction peaks were observed in the XRD pattern. The secondary phase of Bi_2_S_3_ was more dominant at higher concentration of Bi in MoS_2_/Bi_2_S_3_ nanocomposites.^41–43^

**Fig. 3:**
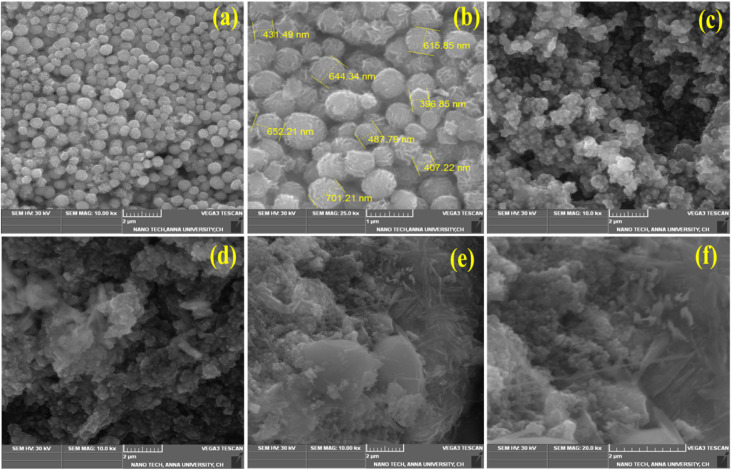
(a and b) SEM images of the pristine MoS_2_, (c–f) SEM images of MBS-1, MBS-2, MBS-3, and MBS-4 samples.

Fig. 3(a) represents the SEM image of pristine MoS_2_, formed as a 3D flower-like nanostructure. The close view of the pristine MoS_2_ nanoparticles is shown in Fig. 3(b) with the size of 300–600 nm range. The 3D nanoflowers showed hierarchical structures with pores and clumsy sheets. The spherical structure was formed due to the agglomeration of more nanosheets together. The morphology of MoS_2_/Bi_2_S_3_ nanocomposites with different concentrations of Bi is shown in Fig. 3(c–f), respectively. Bi_2_S_3_ was grown with MoS_2_ while increasing the Bi concentration in the MoS_2_/Bi_2_S_3_ nanocomposites and clearly observed in the SEM images. The spherical structure of MoS_2_ was stacked over Bi_2_S_3_ nanoflakes in MoS_2_/Bi_2_S_3_ nanocomposites.

**Fig. 4:**
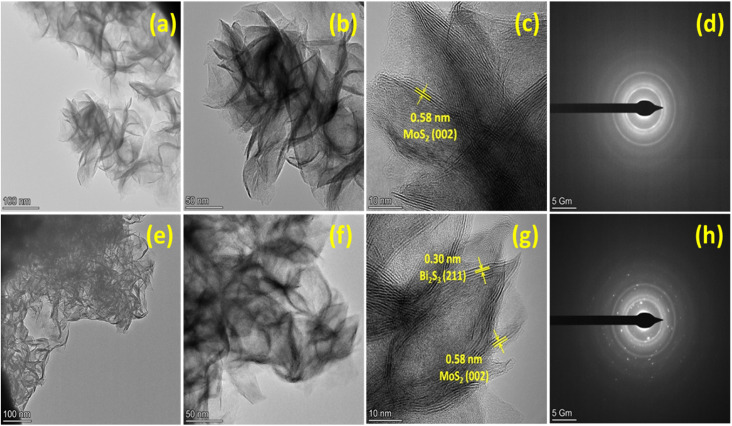
(a–c) HRTEM images of MoS_2_, (d) SAED pattern of MoS_2_, (e–g) HRTEM images of MBS-4 nanocomposite, (h) SAED pattern of MBS-4 nanocomposite.

The TEM and HRTEM were used to analyze the morphology and crystalline nature of the pristine MoS_2_ and MoS_2_/Bi_2_S_3_ nanocomposite and the images are shown in Fig. 4(a–c). The agglomerations of nanosheets were observed and the lattice fringes were clearly observed in the HRTEM images. The interplanar distance of 0.58 nm was calculated from the lattice fringes, which corresponds to the (002) plane of MoS_2_ as observed from the XRD pattern. In Fig. 4(d), the rings corresponding to (002), (100), and (110) planes were observed in the SEAD pattern, which verified the structure of pure MoS_2_ as hexagonal.^44–46^Fig. 4(e–g) represents the TEM and HRTEM images of MBS-4 nanocomposite. It was clearly observed from the images that MoS_2_ and Bi_2_S_3_ were stacked together. The interplanar spacings of 0.58 nm and 0.30 nm were observed, which corresponded to the (002) plane of MoS_2_ and (211) plane of Bi_2_S_3._ The SAED pattern shows a clear ring with spots, which confirms the polycrystalline nature of the MBS-4 nanocomposite (Fig. 4(h)).^47^

**Fig. 5:**
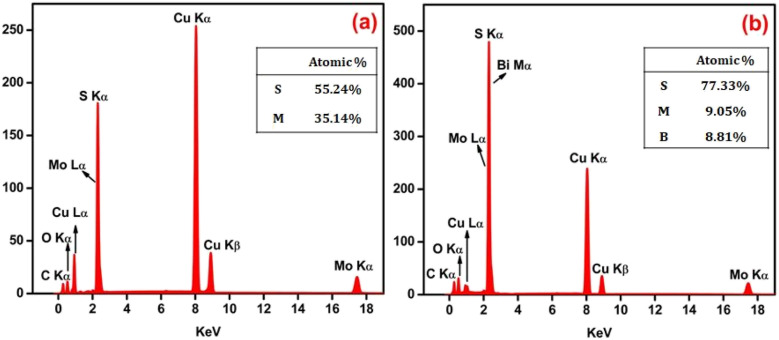
(a and b) EDAX spectra of the pristine MoS_2_ and MBS-4 nanocomposite.

The EDAX spectra were recorded to investigate the composition of the synthesized pristine MoS_2_ and MoS_2_/Bi_2_S_3_ nanocomposite samples. The chemical composition of pristine MoS_2_ was confirmed and displayed in Fig. 5(a). Despite the peaks indicated, copper and carbon peaks were also noticed in the EDAX spectra of both samples, which may have originated from the grid. Fig. 5(b) represents the EDAX spectrum of the MBS-4 nanocomposite, in which the elements of Mo, Bi, and S were clearly observed. The EDAX spectrum confirms that no other elements were present in the sample, indicating that it was homogeneous in terms of its elemental composition.^48,49^

### 3.2. X-ray photoelectron spectroscopy (XPS) analysis

**Fig. 6:**
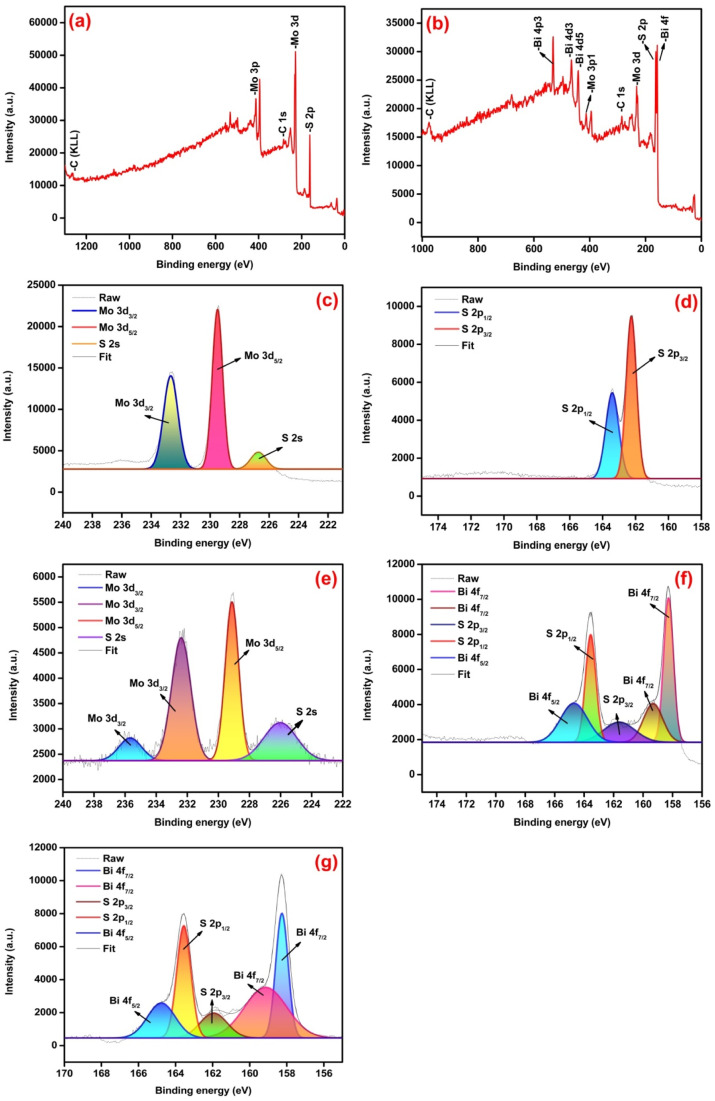
(a and b) Survey spectrum of pristine MoS_2_ and MoS_2_/Bi_2_S_3_ nanocomposite, (c and d) XPS spectra of pristine MoS_2_ for Mo 3d and S 2p respectively, (e–g) XPS spectra of MBS-4 nanocomposite for Mo 3d, S 2p and Bi 4f, respectively.

X-ray photoelectron spectroscopy (XPS) was used to analyze the chemical composition and binding state of elements in the samples. The survey spectra of pure MoS_2_ and MBS-4 samples confirmed the presence of Mo, Bi, S, and C elements as shown in Fig. 6(a) and (b). The elemental carbon appeared as a result of the carbon tape used to hold the samples.^50^Fig. 6(c) represents the core level XPS spectrum of a pristine MoS_2_ sample for Mo 3d. The strong doublet peaks exist at 229.5 eV and 232.7 eV corresponding to Mo 3d_5/2_ and Mo 3d_3/2,_ respectively, and the mild peak at 226.8 eV belonged to S 2s. Fig. 6(d) shows the core level XPS spectrum of S 2p for pristine MoS_2_ sample with two strong peaks appearing at 162.2 eV and 163.4 eV corresponding to S 2p_3/2_ and S 2p_1/2,_ respectively.^51^

Fig. 6(e) represents the core level XPS spectrum of Mo 3d peaks for the MBS-4 nanocomposite. The strong doublet exists at the energy levels of 229.1 eV and 232.3 eV corresponding to Mo 3d_5/2_ and Mo 3d_3/2,_ respectively. Moreover, one of the weaker peaks exists at 226.0 eV for S 2s, and the other is located at 235.8 eV corresponding to Mo3d_3/2,_ which is attributed to the presence of the Mo 6^+^ oxidation state. Oxidation occurred due to the formation of Bi_2_S_3,_ which created the lack of sulfur atoms to form MoS_2._ Such oxidation in the nanocomposite enhances the electronic property of the electrode material and further develops more active sites with good photocatalytic behavior indicating that MoO_3_ is formed in the Bi-substituted nanocomposites.^52,53^

Fig. 6(f) represents the core level XPS spectrum of S 2p peaks for the MBS-4 nanocomposite. The strong doublet peaks appeared at the binding energy of 161.5 eV and 163.5 eV, which correspond to S 2p_3/2_ and S 2p_1/2,_ respectively. Fig. 6(g) represents the core level XPS spectrum of Bi 4f peaks for the MBS-4 nanocomposite. The doublet peaks existed for Bi 4f_7/2_ at the binding energy of 158.2 eV and 159.2 eV, indicating the presence of Bi_2_O_3_ in the composite.^54,55^ The peak corresponding to Bi 4f_5/2_ appeared at the binding energy of 164.8 eV. It is clearly noted that an energy gap of 5.6 eV existed between the two firm peaks of Bi 4f_7/2_ and Bi4f_5/2,_ indicating the formation of the Bi^3+^ state in the Bi_2_S_3_ phase.^56^

### 3.3. Electrochemical analysis

Cyclic voltammetry analysis was used to study the oxidation and reduction reaction kinetics of the pristine MoS_2_ and MoS_2_/Bi_2_S_3_ nanocomposites. The response of the CV curve was significant in the potential window range from −1 to 0 V for all the scan rates from 5 to 100 mV s^−1^. The specific capacitance of active materials can be calculated using the following eqn (1),^57^1
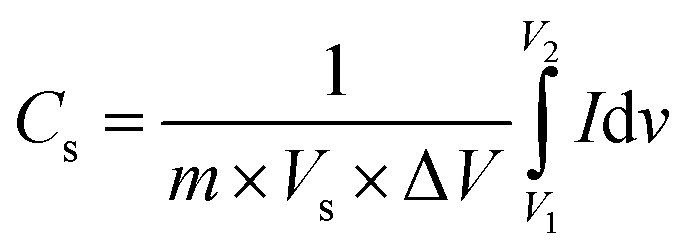
where *m* is the mass of the active material (g), *V*_s_ is the scan rate of the electrode system (mV s^−1^), and Δ*V* is the operating potential window (V).

**Fig. 7:**
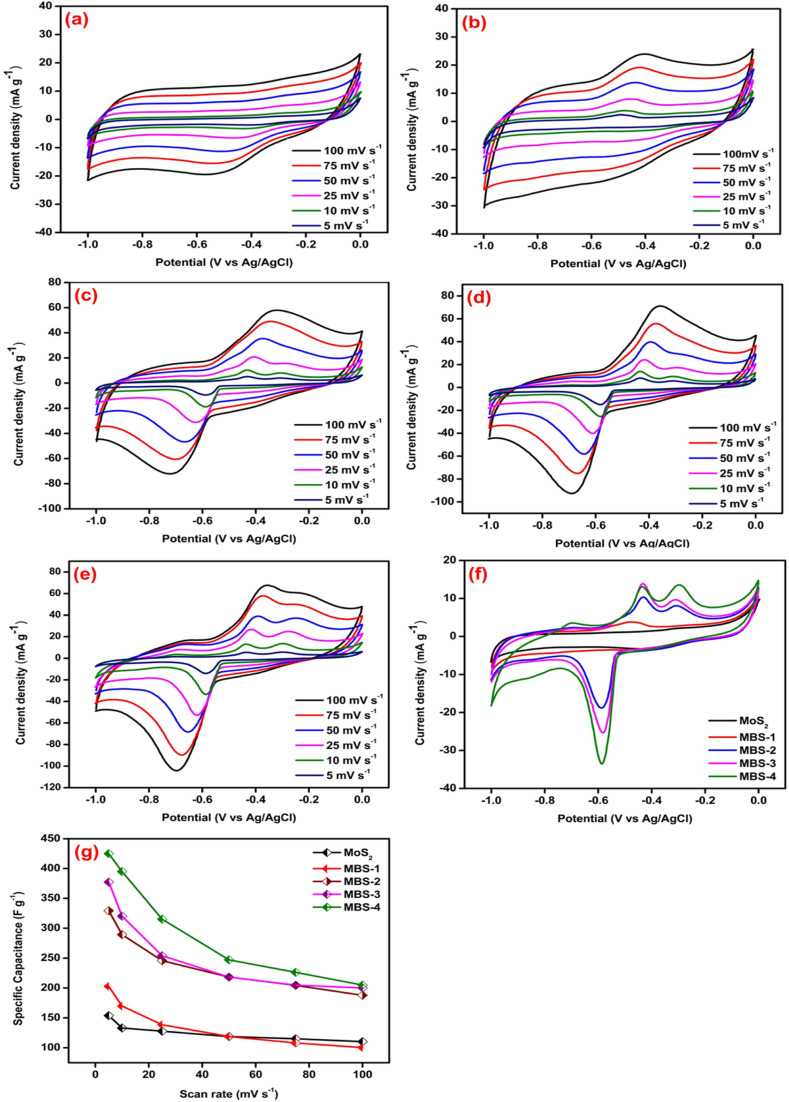
(a–e) Cyclic voltammetry curves of pristine MoS_2_, MBS-1, MBS-2, MBS-3, and MBS-4 electrodes, respectively (f) comparison of CV curves of pristine MoS_2_ and MoS_2_/Bi_2_S_3_ nanocomposites at a scan rate of 5 mV s^−1^, (g) specific capacitances of pristine MoS_2_ and MoS_2_/Bi_2_S_3_ nanocomposites at various scan rates by cyclic voltammetry analysis.

Fig. 7(a–e) shows the CV curves of pristine MoS_2_ and MoS_2_/Bi_2_S_3_ nanocomposites at scan rates from 5 to 100 mV s^−1^. Fig. 7(a) represents the CV response of pristine MoS_2_. The redox peaks are clearly visible in the CV curve, indicating the pseudo-capacitive behavior due to a reversible surface redox reaction.^58^ When the scan rate increases from 5 to 100 mV s^−1^, the area of CV curves also increases and the shape of the curves remains symmetrical. It showed the ideal capacitive behavior of the pristine MoS_2._ The maximum specific capacitance of pristine MoS_2_ was 154 F g^−1^ at a scan rate of 5 mV s^−1^. The CV curves of MoS_2_/Bi_2_S_3_ nanocomposites showed relatively stronger redox peaks compared to those of the pristine MoS_2_, indicating that the Bi-substitution increases the reversible faradaic redox reaction. Fig. 7(b) represents the CV response of the MBS-1 electrode, which exhibited a larger current flow than the pristine MoS_2_. The formation of heterostructure between MoS_2_ and Bi_2_S_3_ increases the conductivity and electrochemical properties of MoS_2_/Bi_2_S_3_ nanocomposites. The highest specific capacitance for the MBS-1 electrode was 203 F g^−1^ at a scan rate of 5 mV s^−1^. The current densities are maximum at higher scan rates due to the rapid diffusion and absorption of ions at the electrode–electrolyte interface.

Fig. 7(c and d) represents the CV curves of MBS-2 and MBS-3 electrodes, respectively. The electrochemical properties of the composites gradually increased with Bi-substitution, as shown in the CV curves. The CV response of the MBS-4 electrode is shown in Fig. 7(e). The two strong oxidation peaks are clearly visible at low scan rates, which correspond to the multiple oxidation states of the elements Mo and Bi. The presence of multiple oxidation states increases the electrochemical redox reactions and conductivity of MBS-4 nanocomposite electrodes. The highest specific capacitance of 425 F g^−1^ was obtained for Mo_0.8_Bi_0.2_S_2_ nanocomposite at a 5 mV s^−1^ scan rate. Fig. 7(f) shows the CV comparison of the pristine MoS_2_ and MoS_2_/Bi_2_S_3_ nanocomposites at a scan rate of 5 mV s^−1^. The MBS-4 nanocomposite has a higher integrated CV area, indicating that more ion diffusion and absorption occur at the electrode/electrolyte interface. It has a strong reduction peak at −0.6 V and two oxidation peaks at −0.42 V and −0.3 V. Fig. 7(g) shows variations in the specific capacitance of pristine MoS_2_ and MoS_2_/Bi_2_S_3_ nanocomposites at different scan rates. The MBS-4 sample had the highest specific capacitance than other composites and pristine MoS_2_. The specific capacitances of the pristine MoS_2_ and MoS_2_/Bi_2_S_3_ nanocomposites were higher at a scan rate of 5 mV s^−1^. At higher scan rates, the ions present in the electrolytes move very fast, which cannot access all the active sites of the electrode surface. This attributes low specific capacitance at higher scan rates.^59,60^ The redox reactions of a MoS_2_/Bi_2_S_3_ composite electrode in the KOH electrolyte can be described as follows:

2 2MoS_2_ + 6OH^−^ → 2Mo(OH)_2_ + 4S^−^_2_ + 3H_2_O + 6e^−^

3 2Bi_2_S_3_ + 6OH^−^ → 2Bi(OH)_3_ + 3S_2_O^2−^_3_ + 6e^−^

In the present work, the ions present in the electrolytes strongly interacted with the active sites of MoS_2_ and Bi_2_S_3_ in the MoS_2_/Bi_2_S_3_ nanocomposite electrodes. Hence, at low scan rates, MoS_2_/Bi_2_S_3_ nanocomposites possess higher specific capacitance. The specific capacitances of pristine MoS_2_ and MoS_2_/Bi_2_S_3_ nanocomposites were calculated at different scan rates using eqn (1) and are listed in Table 1.

**Table 1:** The specific capacitances of the pristine MoS_2_ and MoS_2_/Bi_2_S_3_ nanocomposites at different scan rates by cyclic voltammetry analysis

Sample	Specific capacitance (F g^−1^) at different scan rates					
	100 mV s^−1^	75 mV s^−1^	50 mV s^−1^	25 mV s^−1^	10 mV s^−1^	5 mV s^−1^
MoS_2_	110	115	119	128	133	154
MBS-1	100	108	119	139	170	203
MBS-2	188	204	218	246	289	329
MBS-3	200	205	218	254	320	377
MBS-4	205	226	247	315	395	425

Studying the various kinetic responses through electrochemical analysis enhances the comprehension of the charge/discharge process, and offers valuable insights for pinpointing and developing superior electrode materials for advanced energy storage devices. The capacitive distribution can be approximated using the power law relationship between the current (*i*) and sweep rates (*v*), as shown in eqn (4). This can be performed by determining the *b*-value from the linear slope of the logarithm of *i versus* the logarithm of *v*.

4 *i* = *av*^*b*^

**Fig. 8:**
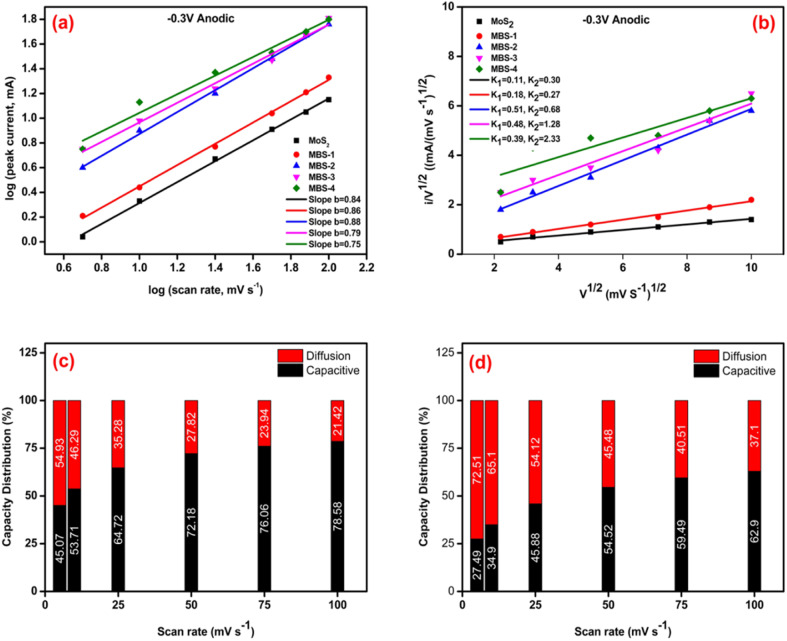
(a–d) Capacitive charge storage distribution calculation, (a) power law (*i* = *av*^*b*^) dependence of current on sweep rate for MoS_2_ and MoS_2_/Bi_2_S_3_ composites, (b) response of *v*^1/2^*vs. i*/*v*^1/2^ (c) comparison of the capacitive and diffusion capacities distribution for MoS_2_ electrode, (d) comparison of the capacitive and diffusion capacities distribution for MBS-4 electrode.

The *b* value has practical applications in designing high-performance electrode materials. It can be used to differentiate between pseudocapacitive and battery-type materials and provide information about electrochemical reactions and charge storage mechanisms in different ion intercalation batteries. In accordance with the existing literature, the *b*-value is characterized by two predetermined values, specifically 0.5 and 1.0, which are indicative of two distinct conditions: diffusion control and capacitive response, respectively. The range of *b* values between 0.5 and 1.0 represents the “transition” area between capacitive materials (*b* = 1.0) and typical battery-type materials (*b* = 0.5).^61–63^Fig. 8(a) shows the *b* values of MoS_2_ and MoS_2_/Bi_2_S_3_ composites. The value of *b* ∼ 0.8 indicates the capacitive behavior of the pure samples. While increasing the Bi concentration is likely favorable with the diffusion of Mo and Bi ions.

J. Wang *et al.* developed a successful approach for analyzing and evaluating the contributions of capacitive and diffusion-controlled processes in the overall current.^64^ By utilizing their calculation method, it became possible to separate and quantify the surface capacitive and diffusion-controlled effects in the current response, as expressed in eqn (5) provided.

5 *i*(*v*) = *k*_1_*v* + *k*_2_*v*^1/2^ = *i*_capacitive_ + *i*_diffusion_

The equation can be transformed into a simpler form known as a first-order equation.

6 *i*(*v*)/*v*^1/2^ = *k*_1_*v*^1/2^ + *k*_2_

By plotting the square root of the relationship between the sweep rate and currents, one can find the values of *k*_1_ and *k*_2_ based on the slope and *y*-axis intercept. The *k*_1_ and *k*_2_ values are estimated from Fig. 8(b). The capacity distribution of MoS_2_ and MBS-4 electrodes are shown in Fig. 8c and d, respectively. MoS_2_ showed 45%, 53%, 64%, 72%, 76%, and 78% capacitive distributions at the scan rates of 5, 10, 25, 50, 75, and 100 mV s^−1^, respectively. The diffusion current was more at the higher concentration of Bi in the MoS_2_/Bi_2_S_3_ composite. The MBS-4 electrode showed 27%, 34%, 45%, 54%, 59%, and 62% capacitive distributions at the scan rates of 5, 10, 25, 50, 75, and 100 mV s^−1^, respectively.

**Fig. 9:**
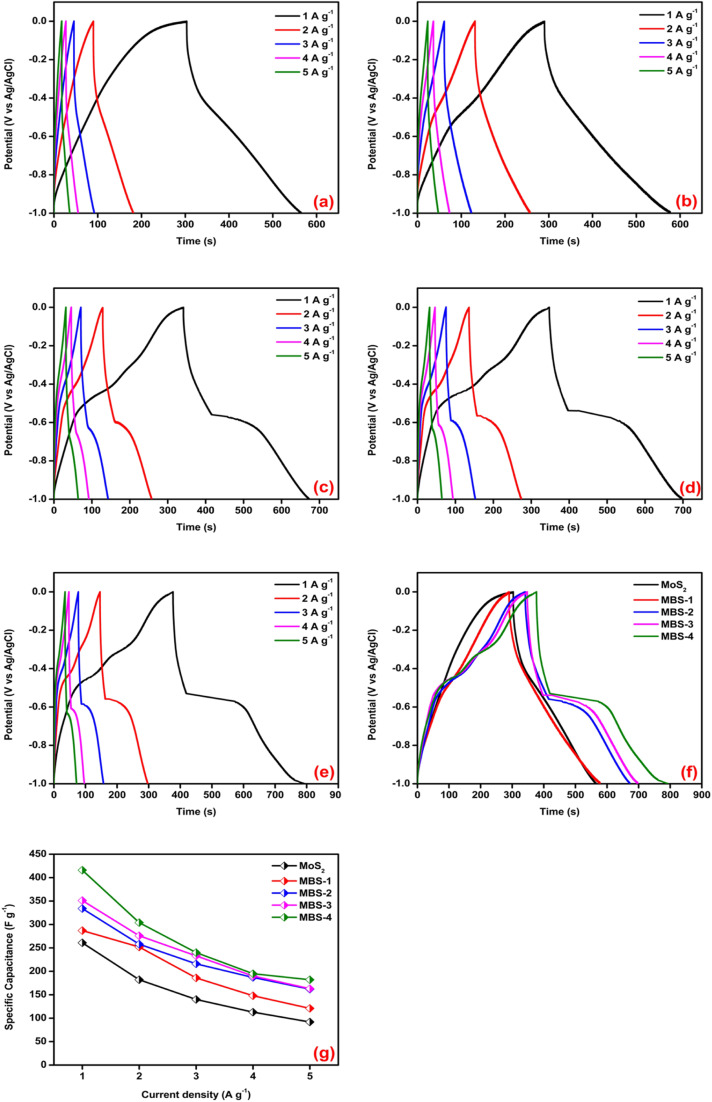
(a–e) Charge–discharge characteristics of pristine MoS_2_, MBS-1, MBS-2, MBS-3, and MBS-4 nanocomposites, respectively, (f) comparison of charge–discharge characteristics of pristine MoS_2_ and MoS_2_/Bi_2_S_3_ nanocomposites at a current density of 1 A g^−1^, (g) specific capacitances of pristine MoS_2_ and MoS_2_/Bi_2_S_3_ nanocomposites at various current densities by charge–discharge analysis.

The charge–discharge kinetics of pristine MoS_2_ and MoS_2_/Bi_2_S_3_ nanocomposites were analyzed using chronopotentiometry. Fig. 9(a–e) shows CP curves of pristine MoS_2_ and MoS_2_/Bi_2_S_3_ nanocomposites at a constant potential window from −1 to 0 V. CP analysis was performed at various current densities from 1 to 5 A g^−1^. The sample discharge time is one of the essential key factors for estimating the specific capacitance of the working electrodes. The specific capacitance of the sample was calculated using the following eqn (7).^65^7
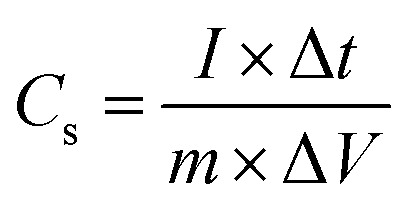
where *m* is the mass of the active material (g), Δ*V* is the stable operating potential window (V), *I* is the current density (A), and Δ*t* is the discharge time (s).

Fig. 9(a) represents the charge–discharge characteristics of pure MoS_2._ The CP curves of pure MoS_2_ are linear and well-symmetrical in shape. The maximum specific capacitance of pure MoS_2_ is 261 F g^−1^ at a current density of 1 A g^−1^. The CP curves of the MBS-1 electrode are shown in Fig. 9(b). The variations in terms of charging-discharging time are clearly observed in MoS_2_/Bi_2_S_3_ nanocomposite electrodes compared to those in pristine MoS_2_. The bismuth substitution increases the reversible charging-discharging performance and it was clearly observed in the CP curves of MBS-2 and MBS-3 electrodes, as shown in Fig. 9(c and d). The specific capacitances are notably increased, indicating more interaction between the active sites of the electrode and electrolytes.

Fig. 9(e) represents the CP curves of the MBS-4 electrode. The remarkable variations are noted in the charging–discharging time of the electrode. The highest specific capacitance of 371 F g^−1^ was calculated at a current density of 1 A g^−1^ among the other electrodes. The comparison between pristine MoS_2_ and MoS_2_/Bi_2_S_3_ nanocomposite electrodes at a current density of 1 A g^−1^ is shown in Fig. 9(f). The electrochemical activities are higher for the MoS_2_/Bi_2_S_3_ composite electrode than the pristine MoS_2._ For comparison, the specific capacitances of all electrodes were calculated from the discharge time of CP at a current density of 1 A g^−1^, and the data are shown in Fig. 9(g). The specific capacitances of pristine MoS_2_ and MoS_2_/Bi_2_S_3_ nanocomposites were calculated at different current densities using eqn (7) and are listed in Table 2. The calculated specific capacitances were high at lower current densities, which indicates that the diffusion of OH^−^ ions was high at low scan rates/current densities, in which the electrolytes have sufficient time to pass through the internal active sites of the electrode materials. At higher current densities and scan rates, the absorption of electrolyte ions took place at the surface of the electrode materials, which led to a less electrode/electrolyte interface and a lower specific capacitance.^66,67^

**Table 2:** The specific capacitances of the pristine MoS_2_ and MoS_2_/Bi_2_S_3_ nanocomposites at various current densities by chronopotentiometry analysis

Sample	Specific capacitance (F g^−1^) at different current densities				
	1 A g^−1^	2 A g^−1^	3 A g^−1^	4 A g^−1^	5 A g^−1^
MoS_2_	261	182	140	113	92
MBS-1	287	252	186	148	121
MBS-2	334	258	216	187	162
MBS-3	351	276	233	190	163
MBS-4	371	304	240	195	182

The Ragone plot is a useful tool for assessing the performance of electrode materials in supercapacitors. It allows for the evaluation of energy density (*E*_d_, measured in W h kg^−1^) and power density (*P*_d_, measured in kW kg^−1^) using the following equations.^68^

8 *E*_d_ = 0.5*CV*^2^/3.6

**Fig. 10:**
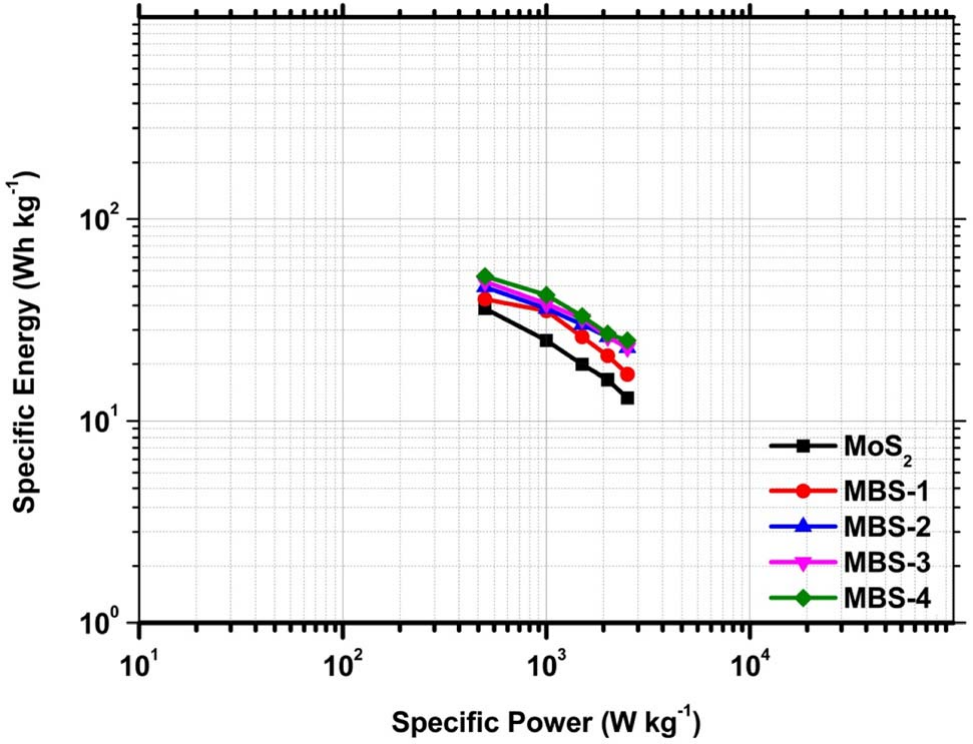
Ragone plot of pristine MoS_2_ and MoS_2_/Bi_2_S_3_ nanocomposites.

9 *P*_d_ = *E* × 3600/Δt

where *C* is the specific capacitance of the electrode (F g^−1^), *V* is the potential change during the discharge process after the internal resistance drop (*v*) and Δ*t* is the discharge time of the electrode (s). The specific energy and power density of pristine MoS_2_ and MoS_2_/Bi_2_S_3_ composite electrodes are shown in Fig. 10.

It was observed that the energy density of the pristine MoS_2_ increases from 13 W h kg^−1^ to 26 W h kg^−1^ at the power densities from 2500 W kg^−1^ to 500 W kg^−1^. Comparatively, the MBS-4 composite electrode shows a higher energy density of 52 W h kg^−1^ at a power density of 500 W kg^−1^ and remained 25 W h kg^−1^ at 2500 W kg^−1^. The results illustrated that the increase in the Bi concentration in MoS_2_/Bi_2_S_3_ enhanced the energy density with high power output.

### 3.4. Electrochemical impedance spectroscopy (EIS) analysis

**Fig. 11:**
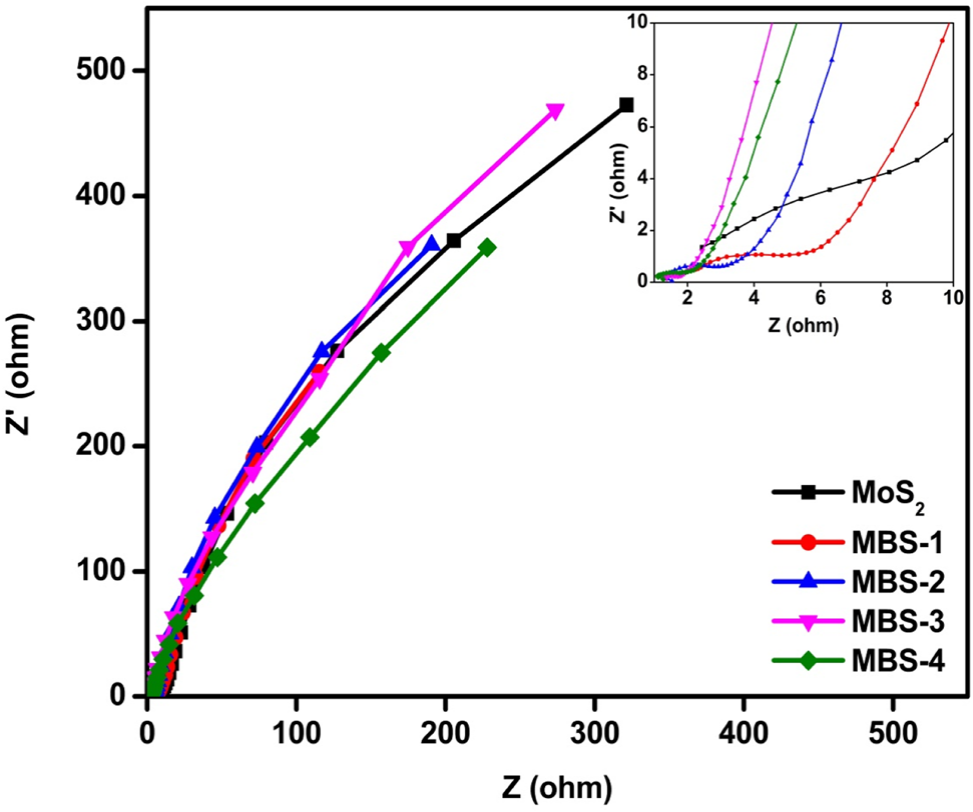
Nyquist plot of pristine MoS_2_ and MoS_2_/Bi_2_S_3_ nanocomposites.

Electrochemical impedance spectroscopy analysis was performed to measure the electrical impedance at the interface between the working electrode and the electrolyte over a wide range of frequencies. Fig. 11 shows the electrical impedance spectra for pristine MoS_2_ and MoS_2_/Bi_2_S_3_ nanocomposite electrodes. It can be observed that a very small semicircle can be observed in the high-frequency range, indicating the low charge transfer resistance between the working electrode and the electrolytes.^69^ In the low-frequency region, the impedance spectrum was linear, confirming the ideal capacitive behaviour of the pristine MoS_2_ and MoS_2_/Bi_2_S_3_ nanocomposites. This indicates a low diffusion resistance between the ions in the electrolytes and the working electrodes. The equivalence series resistance (*R*_esr_) was measured at the intersection of the data with the *x*-axis. The *R*_esr_ of pristine MoS_2_ was 2.4 ohms, which was significantly higher than that of MoS_2_/Bi_2_S_3_ nanocomposite electrodes. The addition of Bi increases the interfacial area between the electrode and the electrolyte and reduces the series resistance, which promoted charge transport. The MBS-4 electrode had the lowest *R*_esr_ among the other MoS_2_/Bi_2_S_3_ nanocomposites. The observed series resistances of MBS-1, MBS-2, MBS-3, and MBS-4 electrodes are 2.2, 1.5, 1.3, and 1.2 ohms, respectively.

## 4. Conclusion

Pure MoS_2_ and MoS_2_/Bi_2_S_3_ nanocomposites with different Bi-concentrations were successfully synthesized using the hydrothermal method. The XRD patterns showed the formation of MoS_2_ and Bi_2_S_3_ phases and the data well matched the standard JCPDS cards. The morphology of synthesized electrode materials was studied using SEM, TEM, and HRTEM analysis. The EDX analysis confirmed the presence of Mo, Bi, and S elements in the synthesized samples and the XPS analysis confirmed the oxidation states of pure MoS_2_ and MoS_2_/Bi_2_S_3_ nanocomposites. The electrochemical properties of MoS_2_/Bi_2_S_3_ nanocomposites were studied using CV, CP, and EIS analysis. MoS_2_/Bi_2_S_3_ nanocomposites showed excellent electrochemical properties with improved electrode/electrolyte interfaces compared to pristine MoS_2._ The calculated specific capacitances of the pristine MoS_2_, MBS-1, MBS-2, MBS-3, and MBS-4 composites were 154 F g^−1^, 203 F g^−1^, 329 F g^−1^, 377 F g^−1^, and 425 F g^−1^ at 5 mV s^−1^, respectively. Electrochemical measurements indicated that the MBS-4 electrode exhibited a higher energy density of 52 W h kg^−1^ than the pristine MoS_2_ electrode (26 W h kg^−1^). The experimental findings suggested that MoS_2_/Bi_2_S_3_ composites showed great potential as high-performance electrode materials in supercapacitors and other energy storage devices.

## Conflicts of interest

The authors declare that they have no conflicts of interest.

## Supplementary Material
